# Staff perspectives on end-of-life care for people living with dementia in residential aged care homes: qualitative study

**DOI:** 10.3389/fpsyt.2023.1137970

**Published:** 2023-04-25

**Authors:** Madeleine L. Juhrmann, Aljon San Martin, Allison Jaure, Christopher J. Poulos, Josephine M. Clayton

**Affiliations:** ^1^The Palliative Centre, Greenwich Hospital, HammondCare, Greenwich, NSW, Australia; ^2^Faculty of Medicine and Health, Northern Clinical School, University of Sydney, St Leonards, NSW, Australia; ^3^Faculty of Medicine and Health, School of Public Health, University of Sydney, Camperdown, NSW, Australia; ^4^Centre for Positive Ageing, HammondCare, Hammondville, NSW, Australia; ^5^Faculty of Medicine and Health, School of Population Health, University of New South Wales, Kensington, NSW, Australia

**Keywords:** dementia, terminal care, end of life decisions, long term care, nursing homes

## Abstract

**Introduction:**

People living with dementia in care homes can benefit from palliative approaches to care; however, not all will require specialist palliative care. The generalist aged care workforce is well placed to provide most of this care with adequate training and support systems in place, but little is known about their experiences.

**Objective:**

To describe staff perspectives on providing quality end-of-life care for people living with dementia in residential care and their families.

**Methods:**

Focus groups and semi-structured interviews were conducted with residential aged care managerial and frontline staff in Australia who were caring for residents living with dementia and end-of life needs. A comprehensive, then snowballing sampling strategy was used in participating care homes. Transcripts were analyzed using reflexive thematic analysis.

**Results:**

Fifteen semi-structured interviews and six focus groups were undertaken with 56 participants across 14 sites across two Australian states. Five themes were identified: putting the resident at the center (creating homes not hospitals, knowing the individual, a case management approach); articulating goals to grant wishes (initiating the conversation, broadening death literacy, avoiding hospitalization); a collective call to action (staffing the home, recognizing deterioration and escalating issues, communication channels and engaging GPs, managing medications, psychosocial supports); educating to empower staff (governance and guidance, mentoring juniors, self-care); and facilitating family acceptance (setting expectations, partnering in care, access at all hours).

**Discussion:**

Aged care staff are committed to providing person-centered palliative and end-of-life care for people living with dementia, recognizing the intrinsic value of each resident, regardless of their declining state. Frontline and managerial staff consider advance care planning, collectively working as part of a multidisciplinary team, access to targeted palliative and end-of-life education and training, and engaging families as key priorities to providing high quality care in care homes.

## 1. Introduction

Global health systems are facing growing pressure to care for people living with chronic diseases as a result of aging populations. Dementia is an increasingly prevalent chronic terminal disease: 55 million people currently live with a formal diagnosis and that is expected to rise to 78 million by 2030 (1). Dementia is associated with functional decline and often requires a tailored physical environment, personal care and behavior management in the case of people displaying behavioral and psychological symptoms of dementia. Progressive deterioration can lead to people requiring full-time care in a residential aged care home (care home) (2). Within Australian settings, more than 50% of people living in care homes have a dementia diagnosis (2). Dementia is the leading cause of death for women in Australia, and second leading cause for men (2).

The World Health Organization defines palliative care as an approach that improves the quality of life of patients and their families who are facing challenges associated with life-threatening illness, through the prevention and relief of suffering through early identification, correct assessment and treatment of other problems, whether physical, psychosocial or spiritual (3). It is well established that people living with dementia can benefit from palliative approaches to care, especially those nearing end-of-life in care homes (4, 5). However, the Royal Commission into Aged Care Quality and Safety has highlighted the significant variation in practice and availability of palliative care within care homes across Australia (6).

A 2014 White Paper provided the first evidence-based definition and priorities of dementia specific palliative care using a consensus approach (7). Literature suggests residents living with advanced dementia would benefit from palliative care that focusses on the management of physical and psychological symptoms, and care home staff need more support from external healthcare services to provide this level of care (8, 9). Dementia specific models of palliative care have emerged in recent years (10–13), including the PACE Steps to Success (14), which identified a generalist and non-disease specific palliative care program for care homes has the potential to improve quality of care and dying in the last month of life for both residents with and without dementia.

Integrating specialist palliative care capabilities into care homes is one approach to address the end-of-life needs of residents living with dementia (15). Yet not all people with dementia in aged care settings will require specialist palliative care, which is an unsustainable approach in isolation, and the generalist aged care workforce is well placed to provide most of this care with adequate training and support systems in place (16).

Previous research suggests clustered domestic residential care homes in Australia result in fewer hospitalizations and better quality of life for residents living with dementia (17). However, a gap remains in the literature on the effect of domestic models of care for residents living with dementia and palliative care needs. Furthermore, little is known about the perspectives of managerial and frontline staff providing end-of-life care to residents living with dementia in care homes (4, 18). These stakeholders offer a unique and practical insight into the complexities of delivering end-of-life care to residents living with dementia, highlighting opportunities for overcoming the challenges and supporting good models of care within this setting. Workforce engagement is also required to help inform and implement best practice palliative and end-of-life care within care homes (14). This study aims to describe staff perspectives on providing quality end-of-life care to be delivered to people living with dementia in residential aged-care homes and their families.

## 2. Methods

We used the consolidated criteria for reporting qualitative health research to report this study (19).

### 2.1. Setting

Within Australia, government funded residential aged care is available for older Australians who are no longer able to live independently in their own home. While models of care may differ between aged care homes, all providers must meet national standards. Participants were eligible if they were a residential care home manager, frontline staff member or volunteer who had been involved with providing palliative and end-of-life care to residents at a large, not-for-profit, Australian residential aged care organization which operates a dementia specific and a predominately clustered cottage model approach to residential aged care (17). The organization’s care homes ranged from 40 to 146 beds, each typically organized into 10–18 bed domestic clusters. Most residents have advanced dementia by the time of admission to the care home.

The organization has adopted a “careworker-led” model of care for residents, supported by a multidisciplinary team that includes residential managers, nurses, volunteers, pastoral care workers, allied health staff and the residents’ general practitioner. Careworkers are frontline staff who provide most of the day-to-day care to residents. This includes personal care, companionship, assistance with feeding and activities of daily living, on-site meal preparation and cleaning. Residential manager roles vary within the organization. Service managers are most often senior registered nurses, overseeing the day-to-day operations of an individual care home. Clinical care managers are nurses who provide clinical oversight of a care home when the service manager is not a nurse. Assistant managers provide support to service and clinical care managers; all of whom report to operational regional managers, who provide oversight for an entire region consisting of multiple care homes. Nurses are frontline staff who provide clinical care, administer restricted medications and liaise with visiting doctors, health professionals, residents’ families and visitors. Volunteers normally visit cottages for short periods of time and provide companionship for residents, including those with palliative and end-of-life care needs. This may include engaging in conversation, games, creative activities and assisting care workers with various activities. Pastoral care workers and some volunteers provide spiritual, religious and emotional support to residents, their families and staff. The organization has an internal mandatory training framework that includes units in dementia and palliative care, to support and equip staff for their unique role.

### 2.2. Participants and recruitment

Staff members were eligible to participate in this study if they were aged 18 years or older and a residential manager, nurse, careworker, pastoral care worker or volunteer involved in the provision of end-of-life care for residents living with dementia. We informed all potentially eligible residential managers about the opportunity to participate in a focus group or individual interview by email invitation and flyers at staff meetings, employing a comprehensive sampling approach. Residential managers who expressed interest in taking part were contacted by the researchers and provided with further information about the study. Following this, a snowballing approach was employed, whereby participating residential managers, as well as volunteer and pastoral care coordinators from the organization, informed potentially eligible frontline staff about the study via email and flyers. Interested frontline staff were then followed up by the researchers. All participants provided written consent to take part in the study. Participant characteristics are outlined in Table 1. The study was approved by the University of Sydney Human Research Ethics Committee (*2018/744*).

**Table 1:** Participant characteristics.

Characteristics	Participants (*n* = 56)
Mean age, y	48
Female, *n* (%)	40 (71)
Country of birth, *n* (%)	
Australia	33 (59)
Role	
Residential manager	20
Nurse	4
Careworker	15
Pastoral careworker	11
Volunteer	6
Level of education	
Completed high school before year 10	2
Completed year 10 of high school	4
Completed final year of high school	5
Professional certificate	12
Undergraduate	27
Postgraduate	6
Formal training in aged care, n (%)	37 (66)
Mean years of experience in aged care, y	10
Training in palliative care, *n* (%)	
No training	6 (10)
On the job training	24 (43)
Short course	21 (38)
Course with qualification	5 (9)
Training in dementia care, *n* (%)	
No training	2 (4)
On the job training	13 (23)
Short course	29 (52)
Course with qualification	12 (21)

### 2.3. Data collection

The semi-structured interview and focus group guides Appendix 1) were informed by a literature review and discussion among the research team, and pilot tested. The questions addressed why they identified certain end-of-life care components important components and how they think end-of-life care could be enabled and improved.

In-person focus groups were organized by staff roles across different geographic networks within the organization, allowing a range of staff from different care homes to participate. Phone interviews were offered to participants who were unable to attend a focus group to obtain more comprehensive data Appendix 2). Two female health researchers unknown to participants, AT (PhD) and CK (MD, PhD), conducted the focus groups. Another two female health researchers unknown to participants, AR (MD) and JB (MD), conducted the telephone interviews between December 2018 and May 2019. All research personnel had completed training and were experienced in qualitative research methods.

Participants were informed that the purpose of the focus groups and interviews was to enable research into improving care quality within the homes. Participant recruitment ceased when data saturation was reached. No follow-up interviews were undertaken. Interviews and focus groups were digitally audio-recorded and transcribed. The researchers also took field notes for reflection during data collection.

### 2.4. Data analysis

Drawing on concepts of reflexive thematic analysis (20) and using a social constructivist theoretical framework (21), we employed an inductive approach, coupled with the researchers’ existing knowledge, to generate codes and identify themes within the data. All the transcripts were inductively analyzed by a female health researcher, MJ (MPH), and the data thematically coded line by line by, developing a coding structure that captured a comprehensive picture of end-of-life care provision at the study sites. The conceptualized issues and ideas were grouped into themes and sub-themes, which were regularly discussed among MJ, JC (MD, PhD) and JSM (RN) to address triangulation and enhance the analytical framework. Microsoft Office Word was used to store, organize, and support analysis of the anonymized data.

## 3. Results

A total of six focus groups and 15 individual interviews were conducted between December 2018 and May 2019 with a total of 56 participants, including: 20 residential managers, four nurses, 15 careworkers, 11 pastoral careworkers, and six volunteers (Table 1). Participants’ work experience in aged care ranged between one and 47 years. The focus groups and interviews ranged in duration between 79–118 min and 37–71 min, respectively. We identified five themes: putting the resident at the center; articulating goals to grant wishes; a collective call to action; educating to empower staff; and facilitating family acceptance. A thematic diagram of the themes and sub-themes is illustrated in Supplementary Figure 1. A table of practical strategies suggested by participants relating to each theme is highlighted in Table 2.

**Table 2:** Strategies suggested by participants for improving end-of-life care for residents living with dementia.

Key areas for improvement	Suggested strategies
Family education and participation	Gently promote family awareness and acceptance of the terminal nature and stages of dementia, death, dying and palliative approaches to care using accessible language.
	Invite families and carers to personalize the resident’s room.
	Ensure 24/7 visitor access to care homes.
	Provide carer support sessions for families and carers to facilitate early bereavement.
Advance care planning	Make advance care planning a national requirement for entry into a care home.
	Revisit advance care planning with the resident (where possible) and/or the family/substitute decision maker soon after admission.
	Support families and substitute decision makers to participate in ongoing advance care planning discussions when the resident no longer has capacity.
Staff experience and education	Provide ongoing opportunities for front line staff to gain practical experience and discuss end-of-life care in a supportive educational manner.
	Mandate essential palliative care experience training for all staff and volunteers, including care home affiliated GPs.
	Develop a palliative care committee of champions in the care home, with representation across all staff roles.
	Ensure staff are adequately supported by pastoral care and/or peer support.
	Enable staff to participate in cultural rituals related to residents dying.
Clinical approaches to care	Ensure all staff can recognize deterioration in the resident and escalate issues to appropriate clinical staff.
	Ensure care homes have adequate clinical back-up support from GPs and linkages to referral pathways for specialist palliative care and/or geriatric services where required.
	Roster regular staffing patterns to promote continuity of care.
	Allocate an extra staff member on shift when a resident is nearing end-of-life.
	Ensure end-of-life medications and equipment are always readily available on site in case of unexpected resident deterioration, and a trained nurse is available to administer.
	Include pastoral care staff in clinical handovers.

### 3.1. Putting the resident at the center

#### 3.1.1. Creating homes not hospitals

Participants emphasized creating *“homelike environments”* (careworker 37), where each resident *“has their own room”* (volunteer 42, residential manager 56) and felt *“comfortable in their own home”* (careworker 38). Participants described personalized environments where *“photos of family are put in the eye view of residents”* (pastoral care worker 47) and scented candles or diffusers were used to *“make the room more peaceful”* (operations manager 56) for the residents and their visitors.

#### 3.1.2. Knowing the individual

The importance of *“respecting the resident’s wishes”* at end-of-life and ensuring no resident got *“out of bed unless they wanted to”* (careworker 35) was emphasized. Some highlighted the need to prioritize person-centered care and fulfill requests at all times of the day and night—*“if they [resident] want a fried egg at midnight, they get a fried egg”* (21). Moreover, pastoral careworkers were particularly cognisant of the varying needs of each resident and suggested a tailored approach was required for meeting the preferences of each individual resident. One participant suggested documenting these needs and sharing them among careworkers, especially those unfamiliar with the resident:

*“Not everyone who walks into that room will have a relationship with the resident who’s dying, so sometimes staff might need a little cheat-sheet” (pastoral care worker 30).*

#### 3.1.3. A case management approach

To ascertain the individual preferences of each resident and their families, participants advocated for a *“case management approach”* (service manager 1, assistant manager 16). One assistant manager described this as *“an ongoing assessment process”* (service manager 3) consisting of multidisciplinary staff meeting with families at regular prescribed intervals, and if the resident were to deteriorate in between *“we just bring it earlier”* (16). Another service manager reiterated:

“*All of our care is premised on knowing the person, knowing the individual resident and tailoring care to them, knowing your staff individually, and their strengths and weaknesses, and competency with palliative care. Knowing the families individually and where they’re up to in the journey*” (service manager 3).

Accordingly, this:

“*Relationship-focused model of care empowers staff to build those relationships with other team members, the family and getting to know the resident*” (service manager 1).

Many participants emphasized the importance of eliciting information of past interests from these meetings to help enhance each resident’s individual abilities, even during end-of-life. Incorporating elements of music, voice and touch into the care of residents living with dementia nearing end-of-life were recommended by participants across all staff groups to “*make them feel that we are still involved, we are still in their world, and they are still connected*” (careworker 19).

### 3.2. Articulating goals to grant wishes

#### 3.2.1. Initiating the conversation

According to the clinicians, initiating goals of care and advance care planning discussions within a case management approach required compassionate communication skills and a multidisciplinary team:

*“We empower and try and equip our [careworker] staff at that level to have conversations that might not always be eloquent, and they might not use all the right language. But when there's a familiarity and there's a family feel and that works well, most of those things are covered. Then the registered nurse comes and supports in the background and then the managers come and have a conversation and turn up at different case conferences that we might have” (service manager, 15).*

Participants recognized family engagement as another key facilitator to establishing goals, suggesting that family members had *“to be ready to hear”* (operations manager 55) and *“be heard”* (careworker 21) for advance care planning conversations to be successful.

#### 3.2.2. Broadening death literacy

Participants believed in the need to explain palliative and end-of-life care to families in *“simplified rather than medical terms”* (clinical care manager 2) and avoid euphemisms of dying as a means of promoting death literacy—the practical knowledge needed to plan well for end-of-life—among family members. Pastoral care participants recognized their *“softer presence”* (31) could offer a unique role during these conversations. They advocated for their involvement during palliative and end-of-life care discussions to address the spiritual distress that can sometimes arise from families and residents.

#### 3.2.3. Avoiding hospitalizations

Participants considered hospitalizations, in most cases, to not be in the best interests of residents living with far advanced dementia, as these unfamiliar environments were *“not going to be able to do anything to change the [palliative] situation”* (pastoral care worker 29). Instead, participants reported prioritizing hospital avoidance as a discussion point during the advance care planning conversations, encouraging families to consider the outcome if their loved one were to be transferred:

*“So it's about having a conversation about what do the family hope to achieve by them [the resident] going to hospital and being transparent with what the outcome is likely to be. If that outcome is death regardless of where they are, then reassuring them that where they are they're going to get the best possible care with us” (service manager 10).*

These advance care planning conversations were described by participants as a dynamic and ongoing process, *“built on each time”* and progressing with *“more depth and detail”* (clinical care manager 5) as the resident living with dementia deteriorated to the end-of-life phase.

### 3.3. A collective call to action

#### 3.3.1. Staffing the home

Participants recounted a preference for *“regular staff”* (service manager 3) rostering across all roles to ensure consistency of care. One assistant manager recalled the practice of confirming a nurse from the previous day was always on to make sure *“information is being handed over well”* (18). Other participants discussed the importance of backfilling positions when a resident was dying to ensure a familiar careworker was always *“able to go and sit with that person and know that I’m not leaving two partners short”* (careworker 37). Furthermore, an operations manager suggested without the “*right staff and skills mix you can have all the best intentions, but it does not do much*” (56).

#### 3.3.2. Recognizing deterioration and escalating issues

Careworkers described a unique ability to recognize distress and decline in a resident living with dementia because they were *“at the frontline”* (19) and knew the residents best. However, despite this strong sense of familiarity, non-clinical staff expressed difficulty in responding to deteriorating residents alone, and described a need to be able to escalate issues within the team:

*“A clinical situation in palliative care can be really challenging for us because maybe we are not all competent enough and we are not that knowledgeable. In that case, registered nurses and the managers would be called to come and help us” (careworker 19).*

Effective communication channels were highlighted by participants as a priority for enabling staff members to *“draw on each other”* (operations manager 6) and *“get to know one another’s strengths”* (service manager 3).

#### 3.3.3. Communication channels and engaging GPs

Clinical staff also recognized the importance of using engaging support when an end-of-life scenario arose. Nurse and residential manager participants referred to engaging “Geriatric Flying Squads,” specialist outreach clinical teams based in local hospitals, who provide assessment and clinical care for residents experiencing acute decline who might otherwise require transfer to hospital if not seen by these health professionals. Participants described calling these teams to help minimize hospitalization, “*because general practitioners do not always come overnight*” (clinical care manager 9) and often hesitated when prescribing end-of-life medications. However, despite clinical staff sometimes reporting challenges when engaging general practioners, all participants still considered them to be an integral part of the team:

*“Getting towards the end stage, it's about having the general practitioner responsive at that point, coming in and making sure that we're all on the right page and what we're seeing is correct and then supporting that for us, being available to talk to the family, if they wish, but also being prepared to provide us with the scripts for sub-cut morphine and things like that if needed” (operations manager 55).*

#### 3.3.4. Managing medications

Participants also recommended a whole-team approach for managing the medication needs of residents living with dementia, highlighting the benefits of clinical staff being well prepared and stocked with *“the right medications and methods of administration”* (service manager 10) for when residents deteriorate. One nurse recounted contacting pharmacists *“three months ahead of time”* to ensure adequate dosages of *“morphine is already in place”* (49). An operations manager cited the value of clinical staff being familiar with palliative medication routes of administration, including *“syringe drivers being available and staff knowing how to use them”* (56). Clinical staff described weekly clinical issues meetings as an imperative opportunity to de-prescribe inappropriate medications and “*think about what’s happening for that [end-of-life] resident to help inform conversations about what to do next*” (clinical care manager 11) to help reduce end-of-life symptoms while addressing polypharmacy.

#### 3.3.5. Psychosocial supports

Staff described responding to the spiritual and existential distress of end-of-life residents living with dementia and their loved ones, bringing them “*peace and comfort, aside from just meeting their medical needs*” (service manager 1). Pastoral care workers were considered an essential component of comprehensive end-of-life care by other participant groups, harnessing unique skills that could make the experience a “*very calm and peaceful time, enabling families to connect and understand what’s going on*” (operations manager 56). According to participants, pastoral care worked in conjunction with the frontline staff and was inclusive to all residents living with dementia and their families, regardless of their religiosity:

*“To those that have always declared no faith, don't believe in anything, or their family have said no, they've never been of a faith. Then I support them, I acknowledge them through what they've done in life, if they've been involved in a particular community service, or an organisation, or they've supported something. Everybody has always done something amazing within their communities, so I'll zone in on that, and just sing their praises” (pastoral care worker 46).*

A clinical care manager acknowledged the emotional impact of caring for palliative and end-of-life residents living with dementia had on the team, suggesting pastoral care and psychosocial support was *“not just for the family and the resident, but for staff as well”* (5).

### 3.4. Educating to empower staff

#### 3.4.1. Governance and guidance

Participants reflected on the utility of clear governance and guidance to facilitate high quality care. A blended model of both *“theoretical and hands on training”* (pastoral care worker 27) was recommended by participants to ensure different learning styles were being catered for. Managers and nurses highlighted their appreciation for team-based education sessions, whereby staff had the opportunity to *“talk through what made them uncomfortable”* (service manager 15) and problem-solve complex situations among one another to normalize uncertainties. One service manager recalled observing and experiencing challenging advance care planning conversations, which prompted her to facilitate multidisciplinary training within her service, including workshopping “*potential responses we can give to the families in the event they ask these tricky questions*” (10). Another clinical care manager employed an end-of-life care flipchart “*that goes through the basics of care*” for nurses to use as a supportive tool for training careworkers, highlighting *“what to expect in every scenario, to be able to provide mouth care, eye care, whatever it may be”* (18). Participants also remarked on the need to train volunteers and *“not just throw them in there”* (pastoral care worker 47). One volunteer conceded, *“in order to provide spiritual care to someone with late-stage dementia, to be most effective you need to have some knowledge of dementia”* (42).

Participants recognized gaps in current training regimes and suggested opportunities for improving future education programs. One careworker emphasized the importance of embedding person-centered care into education, challenging the need to main a rigorous adherence to task-oriented care at the end-of-life:

*“Staff need training if the resident refuses showering for two, three days or four days, that's fine - let them refuse. At least, it can make a peaceful environment for that resident at end-of-life” (43).*

Other participants recalled the importance of providing *“ongoing training”* (careworker 41), as high attrition of staff necessitated continual learning opportunities being offered, particularly to frontline workers.

#### 3.4.2. Mentoring juniors

Participants discussed the need to entrench supportive and sustainable structures for all new staff members, highlighting the benefits of mentoring *“new graduate nurses and giving them access to advice”* (service manager 1). One careworker reflected on their own experience, acknowledging *“if you have a great companion who works with you from the start, teaches you and makes you feel at home in the sense of whatever you are doing, I found that I was on top of it”* (21). A clinical care manager relayed opportunities for more learned staff to role model their experiences:

*“Each time you go through end-of-life with somebody it’s building on that experience, and then that experience can help junior staff with providing that support as it may be the first resident they are palliating” (5).*

Participants also considered providing new staff the opportunity to observe a positive and accepting death as an essential tool to successfully integrate staff into the workplace:

*“Bringing new staff along for that journey can help them realise it's an amazing experience. Good death can have such a positive impact on the families and be a great way for them to say goodbye” (operations manager 55).*

#### 3.4.3. Self-care

Participants described leaning on their colleagues, in both a literal and metaphorical sense, to help them grieve after the death of a beloved resident:

*“Once I really needed a shoulder to cry on when the resident passed away and my partner [staff member] just offered hers” (careworker 24).*

Pastoral care worker participants placed particular emphasis on fostering self-caring environments within the workplace, allowing *“staff to be able to express their grief too”* (31). One participant recalled *“gathering the careworkers around to say a prayer, or something that can be done in that space as a pause point and to acknowledge that this person has died, give thanks for their life and for the care that they have received”* (pastoral care worker 30). Frontline staff also reflected on attending funerals of deceased residents allowing staff to seek closure, “*especially if they had a really good relationship beforehand with the family and the resident who just passed away, to say goodbye, it just makes it so much smoother and calmer*” (careworker 44).

### 3.5. Enabling family acceptance

#### 3.5.1. Partnering in care

Participants advocated for partnering in care with families and shared decision-making throughout the end-of-life journey. According to participants, this strategy delivered stronger outcomes for the resident living with dementia at end-of-life, cultivating a foundation of intergenerational acceptance and early bereavement among loved ones:

*“We encourage families not to give up at end-of-life, but to continue to sit with their loved one and to affirm them, and to say what they need to say. It helps families, and I believe it helps the resident too, to form closure” (pastoral care worker 46).*

Participants highlighted the importance of recognizing family needs and preferences to ensure *“everyone in the resident’s circle is being cared for, because if they are worrying about their loved one, they are not content, comfortable or peaceful”* (pastoral care worker 48). Participants reported engaging willing family members in meaningful tasks to assist the resident, especially at end-of-life, to help them feel empowered and connected:

*“Excellence at end-of-life care is also possible where family feel like they are definitely partnering in care and are feeling enabled … washing their clothes or bringing in extra food that they have cooked themselves. Or towards the end of life, being able to clean them, give them mouth care, or hand care. Even after death, if they wish to, or they can be asked if they would like to help with washing the body of their loved one” (careworker 35).*

Many participants recalled demanding moments with families at end-of-life, emphasizing the importance of *“maintaining that relationship and open communication”* (residential manager 7) to overcome adversity. Support groups for carers were raised by multiple participants as a forum for responding to difficult questions, as well as building death literacy among families and carers:

*“Carer support groups, often being led by our pastoral care staff, actually support families to think further down the line about what's going to happen and to talk about it more publicly. It raises an awareness and reduces some of the stigma for people” (operations manager 56).*

However, frontline staff also recommended remaining open-minded to differing levels of family involvement as the end-of-life journey was challenging for everyone:

*“Make sure that you are non-judgemental because a lot of the residents’ families, a lot of them don’t show up, not because they don’t care, but they just couldn’t handle a loved one dying” (nurse 49).*

#### 3.5.2. Setting expectations

Participants recalled occurrences of families resisting end-of-life care, often driven by a disbelief of their loved one’s terminal fate. To address these discrepancies, participants advocated for setting realistic expectations from the beginning and chipping away at denial through meaningful and compassionate conversations:

*“A big signal for me is if they mention that they hope the person will get better and improve. This person is going to need ongoing conversations, and that decline is going to come as a shock, and they’re going to be quite upset” (clinical care manager 14).*

Participants recognized such conversations may need to be *“revisited several times”* (pastoral care worker 26) before a family comes to terms with the terminal nature of dementia. However, participants expressed a need to *“be truthful with the family”* (careworker 40) and lead with vulnerability, noting *“we cry together”* (nurse 45).

#### 3.5.3. Access at all hours

Finally, participants recognized the enduring nature of end-of-life care and fluctuations in a resident’s condition required families to be regularly notified. All participants endorsed families having 24/7 access to the care home, emphasizing *“we do not have visiting hours”* (careworker 19). One residential manager acknowledged the benefits of this arrangement, highlighting the respect this practice engendered to families:

*“Families have all reported how much they feel supported, and they can come and go as they choose, they never feel like a burden” (10).*

Pastoral care worker participants recalled strategies to facilitate after-hours visits, including getting *“lounge chairs in there [resident’s room] so families can sleep over”* (29) and *“making sure they are introduced to the kitchen”* (47) to prepare food when staying out-of-hours. Such measures reportedly enabled families to feel considered and cared for; a sentiment all participants expressed as a priority for high quality end-of-life care for residents living with dementia.

## 4. Discussion

The participants of this study were committed to providing person-centered palliative and end-of-life care for people living with dementia, recognizing the intrinsic value of each resident regardless of their declining state. Frontline and managerial staff considered ongoing advance care planning, collectively working as part of a multidisciplinary team, access to targeted palliative and end-of-life education and training, and engaging families as key priorities to providing high-quality care in these settings.

The Australian Royal Commission into Aged Care Quality and Safety consistently raised concern over the limitations of aged care workforce capacity (6); a sentiment reinforced worldwide by the extreme demands experienced by aged and health care workers during the COVID pandemic (22, 23). The participants of this study emphasized the observed benefits of continuity of care to residents living with dementia and end-of-life care needs, suggesting regular staffing patterns were integral to achieving this. However, difficulty with staff retention, as echoed in the findings from our study, remains an enduring problem for the aged care sector (24). The international health services community ought to prioritize research that will assist in building a sustainable aged care workforce capable of withstanding the pressures of an aging population, recognizing and responding to the increasing prevalence of dementia among the cohort, and delivering high-quality palliative and end-of-life care to these people.

Acknowledging the different assets within a multidisciplinary team and integrating a strengths-based approach to staffing was another key finding of this study. Participants recalled the merits of a multidisciplinary approach to end-of-life care, harnessing the combined interpersonal skills of care workers, volunteers and pastoral care workers with the clinical decision-making capacity of nurses and GPs to achieve good outcomes for residents living with dementia. However, the participants also recognized significant limitations in achieving timely access of primary and specialist palliative care services for clinical in-reach support. Future research should consider how to alleviate this clinical gap. Building stronger and more sustainable partnerships between aged, primary and specialist palliative care services will help address the workforce capacity and capability challenges facing the sector (25); however, it will require targeted implementation research and translation into practice (26).

Participants described a keen desire to keep residents living with dementia away from hospital due to the distress and disorientation it too often caused them. Death in hospital for patients with dementia is also associated with poorer quality of end-of-life care when compared to death in a care home (27). As such, reducing avoidable hospital admissions of palliative residents living with dementia should remain a key priority for care homes. However, facilitating this goal often requires the prescription of vital end-of-life medications: a skill remaining beholden to the omnipresent scarcity of general practitioners and palliative care nurse practitioners in aged care (18, 28, 29), especially after-hours. Our findings propose general practitioners may also express hesitancy in prescribing end-of-life medications to residents living with dementia. Literature suggests Australian general practioners working in aged care lack knowledge around end-of-life law despite frequently making end-of-life decisions in clinical practice (30), potentially contributing to their reluctance to prescribe end-of-life medications. General practitioners could be better supported to provide high-quality palliative and end-of-life care in care homes through specific training initiatives (31) and greater incentives to work out-of-hours (32). Paramedics could also offer adjunct palliative and end-of-life care support to care homes, especially after hours when general practitioners and community palliative care services might not be available, to reduce avoidable hospital admissions (33).

The findings of our study highlighted opportunities for medical staff to deprescribe medications for palliative residents living with dementia during multidisciplinary clinical issues meetings. Participants described instances where polypharmacy could be identified for residents living with dementia nearing ending of life, and action could be taken by clinical staff to reduce this risk. Facilitating continuity of general practice care for new residents, and more structured transfer to new general practioners upon entering a care home, may prevent potentially inappropriate initiation of medications for people living with dementia (34).

Finally, conversations around goals of care and treatment decisions are recurrent throughout the trajectory of a resident living with advanced dementia in a care home. Given residents with advanced dementia usually no longer have capacity to make health care decisions, families and carers are significant stakeholders in this process and should ideally be involved in shared decision making, which can be assisted with the early and ongoing engagement with families and carers around advance care planning (35). However, as our study reported, families often experience distress during these conversations and need access to information and emotional support throughout the dementia journey (36, 37). Furthermore, advance care planning is commonly approached too late, relying upon proxy-decision making in the care home, in contrast to inclusive and timely supported decision-making with the person living with dementia themselves (38, 39).

To expediate person-centered care at end-of-life, proactive approaches to advance care planning, ensuring people living with dementia have the opportunity to express their wishes and preferences before losing capacity (38), ought to be considered gold standard. Yet the reality is that many people with dementia miss out on having these opportunities in the earlier stages of dementia prior to admission to residential aged care, and by the time of admission many residents already have advanced stages of dementia (39, 40). Therefore, residential aged care staff need access to appropriate dementia-specific education and training to initiate advance care planning conversations with families and carers if the resident no longer has capacity to make healthcare decisions, and the ability to conduct palliative care needs assessments where appropriate (41).

### 4.1. Limitations and further research

The participants were from one single aged care provider and model of care in Australia, therefore potentially limiting the transferability of the results within Australia and abroad. We did not note any apparent differences based on the demographic characteristics of the participants. However, a diverse range of roles were included, and their accounts were compared throughout the data analysis process. Further research could investigate the efficacy of differing models of end-of-life care for residents living with dementia in care homes, comparing approaches and economically evaluating a return on investment in each case. Exploring broader perspectives, including those of general practitioners working in residential aged care, as well as families and carers of residents living with dementia, could also be useful in helping shape future models of best practice end-of-life care for residents living with dementia in care homes.

## 5. Conclusion

Care homes are often where people living with dementia spend their last days. The participants of this study expressed a resounding commitment to providing person-centered end-of-life care for residents living with dementia in care homes, regardless of their diminishing capacity to make decisions. However, participants considered advance care planning, multidisciplinary approaches to care, access to palliative and end-of-life care specific education and training and partnering in care with families’ as key priorities for this approach to be successful. This study has identified specific strategies (see Table 2) to enhance the abilities of care home staff to provide high quality end-of-life care to residents living with dementia. When equipped with these tools and strategies, care home staff believed they could provide high quality end-of-life care to residents living with advanced dementia. Findings from this study identified a need for care home staff to have access to appropriate dementia-specific education and training to initiate advance care planning conversations with residents, families and carers; and build capacity of clinical staff to conduct regular palliative care needs assessments for residents. Future research should focus on how to build more sustainable partnerships between aged, primary and specialist palliative care services to address the workforce capacity and capability challenges facing the sector. General practitioners also require dementia-specific palliative care training initiatives and greater incentives to work out-of-hours in residential aged care.

## Data availability statement

The raw data supporting the conclusions of this article will be made available by the authors, without undue reservation.

## Ethics statement

The studies involving human participants were reviewed and approved by the University of Sydney Human Research Ethics Committee (2018/744). All participants provided written consent. The patients/participants provided their written informed consent to participate in this study.

## Author contributions

The search strategy was developed by JC, CP, and AJ. AJ collected the data. MJ conducted the thematic analysis, which was cross-checked by JC and AS, and drafted the manuscript. All authors contributed to the article and approved the submitted version.

## Conflict of interest

The authors declare that the research was conducted in the absence of any commercial or financial relationships that could be construed as a potential conflict of interest.

## Publisher’s note

All claims expressed in this article are solely those of the authors and do not necessarily represent those of their affiliated organizations, or those of the publisher, the editors and the reviewers. Any product that may be evaluated in this article, or claim that may be made by its manufacturer, is not guaranteed or endorsed by the publisher.
