# Supplementary material for: Staff perspectives on end-of-life care for people living with dementia in residential aged care homes: qualitative study
Source: Front Psychiatry. 2023 Apr 25;14:1137970. doi: 10.3389/fpsyt.2023.1137970 (PMC10166813; doi:10.3389/fpsyt.2023.1137970)
Supplement: Supplementary file 1 [file Data_Sheet_1.docx]

Supplementary Material

Staff perspectives on end-of-life care for people living with dementia in residential aged care homes: qualitative study

Madeleine L Juhrmann*, Aljon San Martin, Allison Jaure, Christopher J Polous, Josephine M Clayton

*** Correspondence:** Madeleine Juhrmann: mjuhrmann@hammond.com.au

# Supplementary Figures

Please see Figure 1 Thematic Summary

# Supplementary Appendices

Please see Appendix 1 Interview and focus group guide

Please see Appendix 2 Participant groups


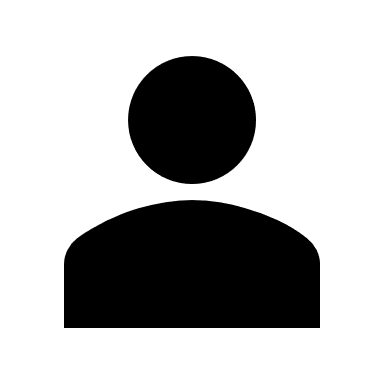
**Figure 1** Thematic summary

**Putting the person at the centre**

- Creating homes not hospitals
- Knowing the individual
- A case management approach

**Articulating goals to grant wishes**

- Initiating the conversation
- Broadening death literacy
- Avoiding hospitalisation

**Educating to empower staff**

- Governance and guidance
- Mentoring juniors
- Self-care

**Facilitating family acceptance**

- Setting expectations
- Partnering in care
- Access at all hours

**A collective call to action**

- Staffing the home
- Recognising deterioration and escalating issues
- Communication channels and engaging GPs
- Managing medication
- Psychosocial supports

**Appendix 1** Interview and focus group guide

**Important components of quality end-of-life care**

1. What does excellent end-of-life care look like to you for people with advanced dementia in residential aged care?
   - What are the most important things needed to provide excellent end-of-life care for people with advanced dementia? Why?
2. What are the barriers to providing these things in your [care home/facility/cottage]? How could these barriers be overcome?

**How to enable delivery of quality end-of-life care**

1. In an ideal world with unlimited resources, can you suggest any improvements that could be made to improve end-of-life care for residents with dementia at your facility?
2. Now, in the real world, what would you prioritise -- Why?
3. Do you have any other suggestions for improvement? Are there any supports, training, resources, or policies that you feel are lacking?
4. Is there anything else that you think might be important or relevant to add?

| **Appendix 2** Participant groups | | |  |
| --- | --- | --- | --- |
| **Data source** | **Participant group** | **Number of participants** | |
| Focus group 1 | Managers | 8 | |
| Focus group 2 | Managers | 8 | |
| Focus group 3 | Careworkers | 7 | |
| Focus group 4 | Pastoral care workers | 7 | |
| Focus group 5 | Careworkers | 6 | |
| Focus group 6 | Volunteers | 5 | |
| Interview 1 | Managers | 1 | |
| Interview 2 | Managers | 1 | |
| Interview 3 | Managers | 1 | |
| Interview 4 | Pastoral care workers | 1 | |
| Interview 5 | Careworkers | 1 | |
| Interview 6 | Volunteers | 1 | |
| Interview 7 | Careworkers | 1 | |
| Interview 8 | Nurses | 1 | |
| Interview 9 | Nurses | 1 | |
| Interview 10 | Pastoral care workers | 1 | |
| Interview 11 | Pastoral care workers | 1 | |
| Interview 12 | Pastoral care workers | 1 | |
| Interview 13 | Nurses | 1 | |
| Interview 14 | Managers | 1 | |
| Interview 15 | Managers | 1 | |
